# 特发性肺纤维化合并肺癌发病机制的研究进展

**DOI:** 10.3779/j.issn.1009-3419.2020.102.23

**Published:** 2020-08-20

**Authors:** 春晖 刘, 元兵 何

**Affiliations:** 830000 乌鲁木齐，新疆医科大学第一附属医院 The First Affiliated Hospital of Xinjiang Medical University, Urumuqi 830000, China

**Keywords:** 特发性肺纤维化, 肺肿瘤, 发病机制, 治疗, Idiopathic pulmonary fibrosis, Lung neoplasms, Pathogenesis, Therapy

## Abstract

特发性肺纤维化（idiopathic pulmonary fibrosis, IPF）是一种原因不明，以弥漫性肺泡炎和肺泡结构紊乱最终导致肺间质纤维化为特征的疾病。针对IPF尚无有效的治疗手段，主要以延缓疾病进展、改善患者生活质量为主。而目前IPF合并肺癌（IPF with lung cancer, IPF-LC）的发病率越来越高，致使患者死亡率明显增加、生活质量显著下降。IPF-LC多见于男性、高龄和吸烟者，是一种临床表现缺乏特异性、无明确治疗方案、中位生存期短、预后较差的致死性疾病。目前IPF-LC发病机制及治疗方案尚不明确。本文就目前IPF-LC的危险因素、发病机制、临床特征和治疗的相关研究进展作简要综述。

## Abstract (en)

Idiopathic pulmonary fibrosis (IPF) is characterized by diffuse alveolitis and disorder of alveolar structure and eventually leads to pulmonary interstitial fibrosis. The cause of IPF is unknown and there is no effective treatment for IPF. There is no effective treatment for IPF, mainly to delay disease progression and improve patient's quality of life. At present, the incidence of IPF with lung cancer (IPF-LC) has increased significantly, which resulted in higher mortality and reduced quality of life. IPF-LC is more common in men, elderly, and smokers. It is a fatal disease and its clinical manifestations lack specificity. IPF-LC has no clear treatment plan, meanwhile, the median survival time is short, and the prognosis is poor. At present, the pathogenesis and treatment plan of IPF-LC are not clear. This article provides a brief review of the current research on the risk factors, pathogenesis, clinical characteristics and treatment of IPF-LC.

特发性肺纤维化（idiopathic pulmonary fibrosis, IPF）被定义为一种病因未明的慢性进行性纤维化性间质性肺炎的特殊表现形式，主要发生于老年人，仅限于肺部发病。IPF的发病率随年龄增长而增加，呈隐匿起病，组织病理学和/或影像学表现为普通间质性肺炎。IPF预后较差，诊断后的中位生存期仅为2年-4年^[1]^，越来越多的研究表明，IPF患者肺癌的发病率较高，发展为肺癌的风险为正常人群的5倍，其发病率从3%-22%^[2]^，在一些案例中甚至超过了50%，其中IPF初次诊断时的年龄、吸烟指数以及IPF诊断后缺乏有效的治疗与肺癌的发生显著相关^[3]^。特发性肺纤维化合并肺癌（IPF with lung cancer, IPF-LC）显著降低了患者的生活质量，增加了患者的死亡率。据报道，IPF患者诊断为肺癌后的1年、3年、5年全因死亡率分别为53.5%、78.6%、92.9%^[4]^。目前的研究表明IPF和肺癌的并存绝不是一种巧合。下面将对IPF-LC的相关研究进展作一综述。

## 1 IPF与肺癌

IPF作为临床上最常见的一种特发性间质性肺炎，其病因不明，治疗上主要以延缓疾病进展，改善患者生活质量为目标。目前临床上越来越多地出现IPF-LC的病例，目前的观点认为IPF本身是肺癌发生的独立危险因素，IPF与肺癌之间的紧密联系表明IPF发病机制的潜在分子变化具有促进肿瘤发生的作用。在有遗传易感性的个体中，原因不明的反复损伤导致成纤维细胞活化、细胞外基质异常累积和肺泡上皮异常细支气管化，从而介导蜂窝囊的形成。累积的基因改变、活化的间充质细胞、化生的上皮细胞之间持续的相互作用促进了癌症的发生和发展^[2]^。若存在高龄、男性、吸烟史、IPF未治疗、用力肺活量快速下降等危险因素时，肺癌发生的风险进一步增加。Ozawa等^[3]^通过对103例初诊IPF时未合并肺癌的患者进行的一项回顾性队列研究证实：年龄、吸烟指数及IPF诊断后缺乏治疗与肺癌的发生有关，在调整了性别因素后，年龄是IPF患者发生肺癌的独立危险因素。类似地，韩国一项纳入938例初诊IPF时未合并肺癌患者的一项回顾性队列研究^[5]^证实：男性、吸烟以及用力肺活量（forced vital capacity, FVC）年下降率≥10%是IPF患者肺癌发生的独立危险因素。由于IPF-LC的临床表现缺乏特异性，因此当IPF患者存在上述危险因素时，我们应当警惕肺癌的发生。国内研究^[6]^发现IPF患者肺癌的发生常常位于肺外周纤维化受累的区域，尤其多见于肺下叶，鳞癌与腺癌是最常见的类型，这与许多国外研究一致，同时该研究还发现IPF-LC患者中胸痛和咯血症状的发生率以及血清肿瘤学标志物癌胚抗原（carcinoembryonic antigen, CEA）和糖类抗原（carbohydrate antigen 125, CA125）水平与单纯IPF患者相比明显升高，这可能对IPF患者早期癌变的诊断具有参考价值。

## 2 IPF-LC的发病机制

### 2.1 遗传变异与IPF-LC

遗传学是指基于基因序列改变所致基因表达水平的变化，如基因突变、基因杂合子丢失和微卫星不稳定性等。大型全基因组关联研究^[7]^表明，有1/3 IPF的发生可以用MUC5B、端粒酶、表面活性物质相关基因的遗传变异来解释。

#### 2.1.1 MUC5B过表达可能是IPF-LC的危险因素

MUC5B是一种主要由黏膜下腺合成的黏蛋白成分，在调节气道炎症、黏液纤毛清除功能、防御肺部感染等方面起着重要作用。在正常情况下终末细支气管及肺泡内并无MUC5B。而在病理情况下，认为MUC5B异常积聚在终末细支气管、肺泡及蜂窝组织内损害了黏膜的宿主防御系统，影响了肺的清除能力以及肺泡的自我修复机制，从而引起肺损伤，最终导致肺纤维化。研究^[8]^表明黏蛋白*MUC5B*启动子rs35705950基因的变异使得MUC5B过表达，IPF患者中MUC5B的表达是非IPF患者的14.1倍，其过表达是发生IPF的重要危险因素。而MUC5B的过表达既与肺腺癌患者的低分化，较高的病理TNM分期和不良预后显著相关，又可以作为诊断肺腺癌的标记物^[9]^，提示MUC5B的表达与肺腺癌之间存在着某种联系。综上所述，MUC5B的过表达与IPF及肺癌的发生发展存在密切联系，因此考虑其异常表达也是促进IPF-LC发病的重要机制。

#### 2.1.2 端粒酶突变所致的端粒缩短可能在IPF-LC发挥作用

端粒是染色体末端的特殊功能结构，在维持基因的稳定性和完整性方面起着重要的作用，端粒功能障碍会导致染色体不稳定及肿瘤的易感性增加。端粒酶逆转录酶（telomerase reverse transcriptase, *TERT*）和端粒酶RNA基因（telomerase RNA gene, *TERC*）是端粒的两个主要基因，在维持端粒DNA的长度、染色体稳定性等方面发挥作用^[10]^。IPF的家族聚集性与*TERT*和*TERC*的突变相关，导致酶活性受损，端粒缩短^[11]^。类似的，对国内IPF患者的研究中也发现*TERT*和*TERC*的突变以及端粒缩短，认为端粒缩短限制了肺泡上皮受到反复损伤后的修复能力，导致瘢痕形成促进了IPF的发展^[12]^，而端粒缩短不仅仅是IPF的危险因素，越来越多的观点认为端粒缩短与肺癌的发生发展显著相关，在一篇纳入了21篇有关端粒缩短与癌症风险之间关系的荟萃分析中得到了同样的结果^[13]^，但其具体机制仍需进一步研究。总而言之，结合上述研究，我们可以推测端粒酶相关的改变可能是IPF-LC的共同致病因素。

#### 2.1.3 p53异常可能导致IPF-LC的风险增加

*p53*基因是到目前为止发现的与人类肿瘤相关性最高的基因，在大约50%的恶性肿瘤中发现*p53*基因的突变，其作为一种抑癌基因，该基因的突变很可能是肿瘤发生的主要发病因素，其突变后患癌的风险增加^[14]^，因为抑癌基因和致癌基因的失衡导致了肺癌的发生。除此以外，抑制p53促使正常成纤维细胞向癌相关成纤维细胞（carcinoma-associated fibroblast, CAF）转变^[15]^，而CAF又是肿瘤基质的重要组成成分，在肿瘤的发生、生长过程中起着一定的作用。抑制*p53*基因又与肺纤维化有关，最新的研究^[16]^表明p53与细胞程序性死亡配体1（programmed cell death 1 ligand, PD-L1）之间有着密切的联系，*p53*基因可能与PD-L1之间存在一个负反馈环，抑制IPF成纤维细胞中的*p53*基因使得PD-L1的表达增加，PD-L1的表达增加进一步促进了IPF肺成纤维细胞的迁移和侵袭能力，而先进的分子技术研究^[17]^发现p53在IPF-LC患者中显著突变。综上所述，推测*p53*的异常在IPF-LC的发生过程中扮演着一定的角色。

### 2.2 信号通路的异常活化与IPF-LC

#### 2.2.1 PD-L1/细胞程序性死亡受体1（programmed death protein 1, PD-1）通路激活引起免疫逃逸导致IPF-LC

PD-L1与IPF存在着密切的联系，在IPF中发现了PD-L1的上调，Geng等^[16]^的研究证实，IPF肺成纤维细胞表达的PD-L1高于健康对照组，而PD-L1的激活显著促进了IPF肺纤维化以及成纤维细胞的侵袭，且可能为侵袭性成纤维细胞表型所必须，借助PD-L1的过表达，侵袭性成纤维细胞驱动进行性纤维化，促进了纤维化的发展，而抑制PD-L1减轻了实验性肺纤维化。同样的，Celada等^[18]^的研究表明PD-1在IPF淋巴细胞上上调，CD4^+^ T细胞的PD1高表达经由信号转导与转录激活因子3（signal transducer and activator of transcription 3, STAT3）信号转导途径增加了白细胞介素17A和转化生长因子-β1（transforming growth factor-beta 1, TGF-β1）的表达从而促进了肺纤维化。此外，PD-L1在多种肿瘤细胞中均表达上调，它与PD-1结合，激活PD-L1/PD-1通路（一条重要的免疫检查点通路），抑制T细胞的增殖和活化，使T细胞处于失活状态，最终被肿瘤细胞用来免疫逃逸^[19]^。PD-1抑制剂和PD-L1抑制剂均可阻断PD-1和PD-L1的结合，上调T细胞的生长和增殖，激活其攻击和杀伤作用，通过调动人体的免疫功能实现抗肿瘤作用，目前已在临床上广泛应用。因此，PD-L1/PD-1通路在促进肺纤维化的同时，肿瘤细胞逃避免疫监视，形成了一个有利于肿瘤发生和生长的微环境，提示该通路似乎是IPF-LC潜在的共同致病机制。

#### 2.2.2 TGF-β信号转导异常在IPF和肺癌发生中的作用

转化生长因子-β（transforming growth factor-beta, TGF-β）作为一种多效性因子，在胚胎发育、细胞分化、器官形成及免疫应答等多种生物过程中发挥调控作用^[20]^。TGF-β信号转导异常在上皮和间充质等变化中均扮演着关键角色，已被证实是肺纤维化和肺癌进展的中心环节^[21]^。目前主要发现3种TGF-β亚型，即TGF-β1、TGF-β2、TGF-β3，其中TGF-β1在纤维化及肺癌相关病理改变中发挥主要作用，而TGF-β/Smad通路是TGF-β发挥生物学效应的主要通路。研究^[22]^表明，TGF-β1经由TGF-β1/Smad2通路诱导A549肺泡上皮细胞（一种保留了肺泡Ⅱ型上皮细胞的重要特征的细胞，被大量用来探索肺泡上皮DNA损伤等研究）发生上皮-间充质转化（epithelial mesenchymal transition, EMT），即上皮细胞向间充质细胞表型转变，表现为E-钙黏蛋白的表达减少，细胞连接蛋白被抑制、上皮细胞的完整性和极性被破坏，而这些病理过程又与IPF纤维化和癌变过程相关。此外，TGF-β1也是肿瘤基质发展的核心，因为TGF-β1也激活了CAF、促进了细胞外基质（extracellular matrix, ECM）的产生，而CAF和ECM又是肿瘤基质的重要组成部分，这形成了有利于肿瘤生长的微环境^[23]^。目前的观点认为TGF-β在癌症发病机理的早期主要作为一种抑制因子抑制细胞的生长，延缓肿瘤细胞的产生，然而在肿瘤细胞出现后，TGF-β通过促进EMT、CAF、ECM的病理过程推动了肿瘤的发展^[24]^。

## 3 IPF-LC患者的潜在治疗前景

目前对于IPF-LC的治疗缺乏共识，无论手术、放疗、化疗都可能诱发IPF的急性加重（acute exacerbation of idiopathic pulmonary fibrosis, AEIPF），从而增加患者的死亡率。随着对IPF和肺癌致病机制的不断了解，尤其是对两者共同致病机制的深入研究，越来越多的临床研究着重于试验具有潜在抗癌、抗纤维化的多效应药物以及传统抗纤维化加抗癌药物的组合在IPF-LC患者中的治疗前景。此外，对早期IPF-LC患者手术术式的合理选择以及术后致死性并发症的预防也是一个值得探讨的话题。

### 3.1 手术治疗

手术是治疗早期肺癌的主要方式，但IPF-LC患者肺切除术后发生AEIPF和死亡的风险较高，Omori等^[25]^报道了IPF-LC组肺癌病理分期为Ia期-IIIa期的以肺叶切除术为主的46例患者中有4例（8.7%）在术后发生了AEIPF，4例患者中的3例（75%）因呼吸衰竭死亡，与对照组相比，IPF-LC组死亡率高，生存率低，术后患者的中位生存期为3.09年，3年和5年总生存率分别为50.9%和22.1%。该研究支持了术后AEIPF的风险会随着切除肺组织体积的增大而增加的观点，经过肺楔形切除术和胸腔镜手术治疗的IPF-LC患者术后未发生AEIPF。同样的，Sato等^[26]^的大样本多中心研究证实，与肺段切除术、肺叶切除术、双肺叶切除术和全肺切除术相比，肺楔形切除术术后间质性肺病急性加重的风险大大降低。此外，在术前的1周-2周口服吡非尼酮600 mg/d，在接下来的1周-2周内，剂量增加到1, 200 mg/d被证实可有效预防IPF-LC患者术后AEIPF的发生率^[27]^。总的来说，手术治疗IPF-LC患者，考虑到肺切除的面积，对早期IPF-LC患者似乎是比较可行的办法，因为相关研究也报道了28例IPF-LC的Ia期非小细胞肺癌患者术后5年生存率为54.2%^[28]^，但仍需综合考虑患者的病情，评估术后AEIPF的风险（如：年龄、AEIPF史、吸烟史、类固醇药物和免疫抑制剂使用史、血清涎液化糖链抗原-6水平、肺功能等）^[26]^、术后肺癌的复发率，恰当地选择合适的术式。

### 3.2 尼达尼布

尼达尼布是一种靶向血管内皮生长因子受体、血小板衍生生长因子受体和成纤维细胞生长因子受体的多重酪氨酸激酶抑制剂，目前已经在临床上广泛用于IPF患者的治疗。而在肿瘤治疗方面，国内多中心Ⅱ期研究^[29]^报道了尼达尼布在一线铂类药物化疗失败的中国晚期（IIIb期以上）的非小细胞肺癌患者中单用尼达尼布的有效性及安全性，结果证实，与厄洛替尼、培美曲塞、多西他赛相比，尼达尼布达到了相似的无进展生存期且耐受性良好。该药还被批准与多西他赛联合使用，尼达尼布200 mg每日2次联合多西他赛75 mg/m²与安慰剂联合多西他赛组相比，肺腺癌患者的无进展生存期明显改善，中位生存期从10.3个月延长至12.6个月^[30]^。综上所述，尼达尼布在IPF和肺癌治疗中均起到了良好的效果，目前仍然缺乏尼达尼布在IPF-LC患者中的相关研究，但基于尼达尼布在IPF和LC治疗中的多效应，期待未来有关尼达尼布治疗IPF-LC患者的相关研究。

### 3.3 化疗

由于化疗可诱发间质性肺疾病的急性加重，因此在化疗药物的选择上应当十分谨慎，尽量选择致间质纤维化作用小的药物，比如卡铂。在18例晚期非小细胞肺癌合并特发性间质性肺炎的治疗中，卡铂联合紫杉醇治疗的有效性及安全性得到了证实^[31]^。2017年在日本开始的为期3年的大样本多中心随机对照研究用于验证尼达尼布联合卡铂以及白蛋白结合型紫杉醇在IPF合并晚期非小细胞肺癌患者中的有效性及安全性，其结果值得期待^[32]^。见表 1。

**1:** IPF和肺癌治疗的相关研究 Research on IPF and LC related treatment

Author (year)	Study design	Patients	Findings
Omori (2015)^[25]^	Retrospective	46 patients with IPF-NSCLC vs 57 patients with IIP-NSCLC (excepted IPF)	The 5-year survival rate was significantly higher in the IIP-NSCLC group (53.2%) than IPF-NSCLC group (22.1%) after pulmonary resection. The MST was 3.09 years after pulmonary resection in the IPF-NSCLC group
Sato (2014)^[26]^	Retrospective	164 patients with ILD-NSCLC. The incidence of acute exacerbation of ILD after pulmonary resection for lung cancer within 30 days	By using the wedge resection group as the referent category, the OR for AE in the segmentectomy group was 3.675, the OR in the lobectomy group was 3.861, the OR in the bilobectomy group was 5.055 and the OR in the pneumonectomy group was 6.953
Iwata (2016)^[27]^	Retrospective	28 patients with IPF-LC treated by pirfenidone during perioperative period vs 72 patients with IPF-LC not treated by pirfenidone during perioperative period	Pirfenidone is an effective and feasible prophylactic treatment to reduce postoperative AEIPF
Saito (2011)^[28]^	Retrospective	28 patients with IPF-LC (Ia NSCLC)	The 5-year survival rate of patients with IPF-NSCLC was 54.2% after pulmonary resection
Dai (2015)^[29]^	Multi-center, prospective	The efficacy and safety of using nintedanib as single-regimen in second-line chemotherapy for Chinese patients with advanced NSCLC	NSCLC patients in second-line chemotherapy reached similar PFS, as compared with other approved second-line regimens and the toxicity of nintedanib was well tolerated
Reck (2014)^[30]^	Multi-center, prospective	655 patients with NSCLC were randomly assigned to receive docetaxel plus nintedanib vs 659 patients with NSCLC were randomly assigned to receive docetaxel plus placebo	Median overall survival 12.6 months in the docetaxel plus nintedanib group vs 10.3 months in the docetaxel plus placebo group
Minegishi (2011)^[31]^	Prospective	18 patients with IIP-NSCLC (6 patients with IPF)	Paclitaxel combination with carboplatin was effective and safe for IIP-NSCLC (advanced). The median PFS, MST and 1-year survival rate were 5.3 months, 10.6 months and 22%, respectively
Otsubo (2018)^[32]^	Multi-center, prospective	Carboplatin plus nab-paclitaxel with or without nintedanib for IPF-NSCLC (advanced)	Ongoing
IPF-NSCLC: idiopathic pulmonary fibrosis with non-small cell lung cancer; IIP-NSCLC: idiopathic interstitial pneumonia with non-small cell lung cancer; MST: median survival time; AE: acute exacerbation; OR: odds ratio; ILD-NSCLC: interstital lung disease with non-small cell lung cancer; IPF-LC: idiopathic pulmonary fibrosis with lung cancer; AEIPF: acute exacerbation of idiopathic pulmonary fibrosis; PFS: progression-free survival.			

## 4 总结

目前的研究表明，IPF-LC的疾病组合在临床上并不是罕见病例，IPF-LC严重影响了患者的生活质量以及生存时间。对于IPF患者，在规律正确的抗纤维化治疗的同时要注意定期随访以便早期观察到肺癌的发生。总而言之，尽管目前对这种疾病组合的治疗缺乏有效的办法，但是通过对其致病机制的深入了解，针对其共同致病机制开发特异性药物或是联合用药可能会是未来有效治疗此疾病的策略之一。
